# Pharmacologic Drug Detection and Self-Reported Adherence in the HPTN069/ACTG5305 Phase II PrEP Trial

**DOI:** 10.1007/s10461-024-04451-7

**Published:** 2024-07-31

**Authors:** Stanley E. Cooper, Shuaiqi Zhang, Daniel Haines, Kenneth H. Mayer, K. Rivet Amico, Raphael J. Landovitz, Craig W. Hendrix, Mark A. Marzinke, Wairimu Chege, Marybeth McCauley, Roy M. Gulick

**Affiliations:** 1 Division of Infectious Diseases, Weill Cornell Medicine, 525 East 68th Street, Baker 24, New York, NY 10065 USA; 2 Fred Hutchison Cancer Center, Seattle, WA USA; 3 Fenway Health, Harvard Medical School, Boston, MA USA; 4 University of Michigan, Ann Arbor, MI USA; 5 University of California, Los Angeles, CA USA; 6 Johns Hopkins University School of Medicine, Baltimore, MD USA; 7 DAIDS, NIAID, NIH, Bethesda, MD USA; 8 Durham, NC FHI 360 USA

**Keywords:** (6/6): Adherence, CASI, HIV prevention, Maraviroc, PrEP, Tenofovir

## Abstract

Adherence drives efficacy in PrEP clinical trials. We compared drug concentrations and self-reported adherence in HPTN069/ACTG5305, a double-blinded, randomized trial of the safety and tolerability of candidate PrEP regimens that included maraviroc (MVC), tenofovir (TDF), and emtricitabine (FTC). Plasma drug concentrations and self-reported adherence by computer-assisted self-interview (CASI) were assessed at study weeks 24 and 48. Descriptive statistics and a generalized linear model were used to assess the association between selected demographic factors, self-report of daily medication adherence and plasma drug concentrations consistent with daily adherence. Among 718 paired observations from 370 participants, 43% (306/718) reported daily adherence by CASI, 65% (467/718) had drug concentrations consistent with daily adherence and 11% (81/718) had CASI responses that reported daily adherence despite having drug concentrations consistent with less-than-daily adherence. In adjusted analyses, participants who were assigned male at birth (aOR 1.42 [95% CI 1.02, 1.97]), older (5-year increments aOR 1.10 [95% CI 1.09, 1.11]), White (aOR 2.2 [95% CI 1.88, 2.56]), had advanced education (aOR 3.89 [95% CI 2.97, 5.09]), were employed (aOR 1.89 [95% CI 1.50, 2.40]), or partnered/married (aOR 2 [95% CI 1.72, 2.32]) were more likely to have drug concentrations consistent with daily adherence. Participants who were not employed (aOR 2.7 [95% CI 1.31, 5.55]) or who were single/not partnered (aOR 2.33 [CI 95% 1.25, 4.34]) were more likely to have drug concentrations that did not reflect daily adherence despite self-reported PrEP adherence. These findings support the need for ongoing adherence counseling in clinical trials of new PrEP regimens.

## Introduction

Medication adherence is critical for preexposure prophylaxis (PrEP) efficacy in oral PrEP clinical trials [1–3]. However, accurate measurements of adherence in the research setting can be difficult to ascertain, as has been shown in several past clinical trials that have demonstrated differences between biomedical indices of medication adherence and self-reported PrEP adherence [4–8]. The discrepancies previously seen between pharmacologic and self-reported indicators of adherence may be narrowing in certain populations, as more recent data from demonstration projects with open-label PrEP suggests greater concordance between pharmacologic measures and self-report [9, 10].

Although self-reported measurements of adherence are convenient as an inexpensive and non-invasive method of measurement, they may be subject to inaccuracies in the setting of recall bias, social desirability, and other factors [2]. A better understanding of predictors of adherence and of the associations between participant characteristics, pharmacologic drug concentrations, and self-reported adherence can help inform the future development of targeted adherence support in clinical trials.

We evaluated drug concentration and self-reported measures of adherence in HPTN069/ACTG5305, a phase II double-blinded, multicenter randomized control trial on the safety and tolerability of maraviroc (MVC)-based daily regimens for PrEP compared to TDF/FTC in at-risk men who have sex with men (MSM), as well as cis and transgender women [11, 12]. This analysis assesses objective pharmacologic-based adherence and subjective self-reported adherence among individuals participating in HPTN069/ACTG5305 and characterizes sociodemographic predictors of PrEP adherence.

## Methods

HPTN 069/ACTG 5305 evaluated 3 candidate PrEP regimens (daily oral MVC alone; MVC + TDF; or MVC + FTC) and 1 control regimen (TDF/FTC) in at-risk HIV-uninfected cisgender men and cis and transgender women aged 18 years or older at twelve US clinical research sites [11, 12]. The study design used matched placebos, such that randomization resulted in all participants taking three pills daily. Participants received one or two antiretrovirals and one or two matched placebos so that all 3 regimens appeared identical. Since this was a phase II study that included candidate PrEP regimens, participants were informed that some of the regimens they might receive had not yet been shown to be effective against HIV transmission. Eligible participants in the current analysis had plasma antiretroviral concentrations as well as behavioral data and self-reported measures of study drug adherence collected by CASI at weeks 24 and 48 of the study.

### Drug Concentration Measure of Adherence

The methods used for classifying plasma drug concentrations as either consistent with daily adherence or less-than-daily adherence were derived from the HPTN 069/ACTG 5305 sub-study on sexual behavior and medication adherence in men who have sex with men (MSM) [13]. Plasma concentrations in three of the four study arms were classified as consistent with either daily or less-than-daily adherence by using established tenofovir (TFV) and FTC benchmarks from the HPTN 066 Directly Observed Therapy (DOT) study [14]. In the MVC + TDF arm, plasma samples were categorized as consistent with daily adherence if TFV concentrations were ≥ 35.5 ng/mL. In the MVC + FTC arm, plasma samples were categorized as consistent with daily adherence if FTC concentrations were ≥ 49.1 ng/mL. For the TDF + FTC arm, plasma samples were categorized as consistent with daily adherence if TFV concentrations were ≥ 35.5 ng/mL and FTC concentrations ≥ 49.1 ng/mL.

For the MVC only arm, plasma samples were considered consistent with daily adherence if MVC concentrations were ≥ 4.1 ng/mL. In the absence of an established MVC benchmark from a DOT study, the MVC drug concentration threshold for daily adherence was estimated by conducting receiver operating characteristics (ROC) analysis on data from HPTN 069/ACTG 5305 participants in the MVC + TDF and MVC + FTC arms. Only for this MVC adherence threshold ROC analysis, samples from MVC + TDF and MVC + FTC arms were classified as high or low adherence using the more stringent median TFV and FTC concentration cut-offs for daily use from HPTN 066 (rather than the more inclusive 90% sensitivity thresholds used in HPTN 066). This ensured that the high adherence group had concentrations clearly consistent with daily drug adherence. Samples from the MVC + TDF arm with TFV ≥ 52.2 ng/mL were classified as daily adherence and were classified as less-than-daily adherence otherwise. Samples from the MVC + FTC arms with FTC ≥ 70.9 ng/mL were classified as daily adherence and less-than-daily adherence otherwise. In each of the MVC + TDF and MVC + FTC subsets separately, ROC curves were used to assess the corresponding range of MVC concentrations consistent with daily drug adherence. The MVC 90% and 100% sensitivity thresholds were determined to be sufficiently similar between the MVC + TDF and MVC + FTC subsets, thus ROC analysis was applied to the combined set of MVC + TDF and MVC + FTC samples to calculate the MVC thresholds of interest. ROC analysis in the combined set estimated a MVC threshold of 4.1 ng/mL for 100% sensitivity. In a follow-up analysis, percent of samples classified as daily adherence was examined across the four study arms. Use of the 4.1 ng/mL benchmark in the MVC only arm was found to yield a percent value that closely corresponded to percentages computed in the other three arms from the HPTN 066 benchmarks. This threshold differed only slightly from a prior analysis (a more stringent 4.6ng/mL) that did not use ROC curves [15].

### Self-Reported Measure of Adherence

Self-reported measures of adherence on study weeks 24 and 48 were evaluated using three CASI items related to adherence.

1. Ability to take study medication every day in the past month (Excellent, Very Good, Good, Fair, Poor, Very Poor).
2. How much of the study drug was taken as recommended in the past month (0–100%, sliding scale).
3. Over the past month, how much of the time was study drug taken as recommended (All of the time, Most of the time, Half of the time, Some of the time, None of the time).

Responses for the three adherence items were linearly transformed to a 0 − 100 point scale for each study participant, with a summary of the individual adherence items calculated as the mean of the three individual items. A composite score equal to or greater than 96 were categorized as daily adherence (reflecting reports of perfect or near perfect adherence on a linearized Wilson 3-item measure summary) [16].

### Statistical Analysis

Descriptive statistics were used to compare the percentage of paired samples with self-reported daily medication adherence and the percentage of paired samples with drug concentrations consistent with daily medication adherence. A generalized linear model controlled for repeated measures was used to estimate adjusted odds ratios (aORs) and 95% confidence intervals (CIs) for associations between sociodemographic characteristics and pharmacologic measures of adherence, as well as for correlates of CASI overestimation of self-reported adherence among paired samples with drug concentrations consistent with less-than-daily adherence. Demographic covariates of interest used in the analysis included sex assigned at birth, age, race/ethnicity, education level, employment status, marital status, report of active illicit drug use, and report of anxiety/depression reported at baseline.

## Results

Among 370 participants enrolled between June 2012 and December 2014 generating a total of 718 study follow-up visits that included both drug concentration and CASI data, 61% were male assigned at birth (227 cisgender men/2 transgender women) and 39% female assigned at birth (140 cisgender women/1 transgender man) (Table 1). The age of included participants ranged from 18 to 70, with median age 32. Nearly half (43%) identified as Black and 18% identified as Latino/Hispanic. A total of 67% reported having some college education or an advanced degree and 29% a high school education or less. About 57% of participants reported illicit drug use during the study period (inclusive of ecstasy, GHB, heroin, non-prescription marijuana, methamphetamine, other hallucinogens, PCP, cocaine, ketamine, tranquilizers, and prescription drugs used recreationally).

**Table 1:** Baseline sociodemographic characteristics of paired observations

Total number of observations	718
Sex assigned at birth	
Male	440 (61%)
Female	278 (39%)
Age at Enrollment	
Median	32
25th, 75th %tile	26, 43
Race	
American indian or alaska native	18 (3%)
Asian	21 (3%)
Black	308 (43%)
Native Hawaiian or pacific islander	6 (1%)
Other	60 (8%)
White	353 (49%)
Ethnicity	
Latino/Hispanic	131 (18%)
Education level	
8th grade or equivalent or less	4 (1%)
Some high school	46 (6%)
High school graduate or equivalent	157 (22%)
Vocational/trade/technical school	28 (4%)
Some college or 2-year degree	215 (30%)
Finished college	187 (26%)
Master’s or other advanced degree	81 (11%)
Employment status	
Full-time employment	302 (42%)
Part-time employment	151 (21%)
Not employed	265 (37%)
Marital status	
Married/civil union/legal partnership	54 (8%)
Living with primary/main partner	117 (16%)
Primary/main partner, not living together	75 (10%)
Single/divorced/widowed	466 (65%)
Other, specify	6 (1%)
Self-reported anxiety/depression	
1. I am not anxious or depressed.	536 (75%)
2. I am moderately anxious or depressed.	162 (23%)
3. I am extremely anxious or depressed.	17 (2%)
Decline to answer	1 (< 1%)
Illicit drug use at baseline	
Missing	4 (1%)
No	304 (42%)
Yes	410 (57%)

Among all included paired samples, 43% had CASI survey responses and 65% had drug concentrations consistent with daily adherence (Table 2). 11% of CASI responses overestimated daily adherence compared to paired drug concentrations. Drug concentrations consistent with daily adherence were more commonly found in samples from those assigned male at birth (72%) than those assigned female at birth (54%) and in samples from White participants (77%) than Hispanic (70%) and Black participants (52%) (Table 3). There was also a stepwise increase in daily adherence by drug concentration with increasing level of education, from some high school or less (44%) to completion of a 4-year college degree (73%) or masters/advanced degree (86%). Samples from married/partnered participants were also more likely to have drug concentrations consistent with daily adherence (73%) than single participants (61%). Participants who reported anxiety or depression at baseline (65%) or who reported baseline illicit drug use (66%) had similar rates of daily adherence as those who did not report anxiety/depression (65%) or illicit drug use (64%).

**Table 2:** Adherence by drug concentration and self-report

	Non-daily Adherence by Self-report	Daily Adherence by Self-report	Total
Drug concentration reflects daily adherence	242	225	467 (65%)
Drug concentration reflects non-daily adherence	170	81	251 (35%)
Total	412 (57%)	306 (43%)	718 (100%)

**Table 3:** Daily adherence by drug concentration measurements

	Non-Daily adherence by drug concentration	Daily adherence by drug concentration
Total number of observations	251 (35%)	467 (65%)
Sex assigned at birth		
Male	123 (28%)	317 (72%)
Female	128 (46%)	150 (54%)
Age at Enrollment		
Median	31	33
25th, 75th %tile	24, 44	27, 43
Race		
American Indian or alaska native	4 (22%)	14 (78%)
Asian	5 (24%)	16 (76%)
Black	149 (48%)	159 (52%)
Native Hawaiian or Pacific Islander	0 (0%)	6 (100%)
Other	24 (40%)	36 (60%)
White	81 (23%)	272 (77%)
Ethnicity		
Latino/Hispanic	39 (30%)	92 (70%)
Education Level		
8th grade or equivalent or less	2 (50%)	2 (50%)
Some high school	26 (57%)	20 (43%)
High school graduate or equivalent	79 (50%)	78 (50%)
Vocational/trade/technical school	9 (32%)	19 (68%)
Some college or 2-year degree	73 (34%)	142 (66%)
Finished college	51 (27%)	136 (73%)
Master’s or other advanced degree	11 (14%)	70 (86%)
Employment status		
Full-time employment	68 (23%)	234 (77%)
Part-time employment	51 (34%)	100 (66%)
Not employed	132 (50%)	133 (50%)
Marital status		
Married/civil union/legal partnership	10 (19%)	44 (81%)
Living with primary/main partner	28 (24%)	89 (76%)
Primary/main partner, not living together	28 (37%)	47 (63%)
Single/divorced/widowed	183 (39%)	283 (61%)
Other, specify	2 (33%)	4 (66%)
Self-reported anxiety/depression		
1. I am not anxious or depressed.	186 (35%)	350 (65%)
2. I am moderately anxious or depressed.	55 (34%)	107 (66%)
3. I am extremely anxious or depressed.	8 (47%)	9 (53%)
Decline to answer	1 (100%)	0 (0%)
Illicit drug use at baseline		
Missing	2 (50%)	2 (50%)
No	109 (36%)	195 (64%)
Yes	140 (34%)	270 (66%)

In multivariable adjusted analyses of drug concentrations of daily adherence controlling for repeated measures, samples from participants who identified as male (aOR 1.42 [95% CI 1.02, 1.97]), were older (by increasing 5-year increments aOR 1.10 [95% CI 1.09, 1.11]), White (aOR 2.2 [95% CI 1.88, 2.56]), had an advanced education (aOR 3.89 [95% CI 2.97, 5.09]), were employed (aOR 1.89 [95% CI 1.50, 2.40]), or partnered/married (aOR 2 [1.72, 2.32]) were more likely to have drug concentrations consistent with daily adherence (Table 4)).

**Table 4:** Odds ratio of drug concentration reflecting daily adherence by sociodemographic characteristics

	Unadjusted ORs (95% CI)	Adjusted ORs (95% CI)
Sex assigned at birth (vs. female)		
Male	2.19(1.91, 2.52)	1.42(1.02, 1.97)
Age (by increasing 5-year interval)	1.05(1.03, 1.06)	1.10(1.09, 1.11)
Race/ethnicity (vs. white)		
Black	0.30(0.26, 0.35)	0.45(0.39, 0.53)
American Indian or Alaska Native, native Hawaiian, Asian or Other	0.54(0.48, 0.61)	0.50(0.48, 0.52)
Hispanic (vs. non-hispanic)		
Hispanic	1.33(1.18, 1.50)	1.28(1.09, 1.50)
Education level (vs. some high school or less)		
High School Graduate or Equivalent	1.27(1.01, 1.60)	1.12(0.87, 1.43)
Vocational/trade/technical school	2.70(1.39, 5.23)	2.52(1.35, 4.72)
Some college or 2-year degree	2.47(2.02, 3.03)	1.54(1.23, 1.95)
Finished college	3.39(3.03, 3.79)	1.57(1.45, 1.69)
Master’s or other advanced degree	8.08(6.28, 10.39)	3.89(2.97, 5.09)
Employment status (vs. no employment)		
Part-time employment	1.94(1.66, 2.28)	1.89(1.86, 1.92)
Full - time employment	3.39(3.10, 3.71)	1.89(1.50, 2.40)
Marital status (vs. married/civil union/legal partnership/primary partner)		
Single/divorced/widowed/Other	0.57(0.47, 0.70)	0.50(0.43, 0.58)
Reports anxiety or depression (vs. denies anxiety and depression)		
Moderately/extremely anxious or depressed	0.99(0.75, 1.31)	1.01(0.86, 1.19)
Illicit drug use (vs. no)		
Yes	1.09(0.85, 1.39)	0.97(0.67, 1.42)

However, when looking at self-reported adherence in participants who had drug concentrations consistent with non-daily adherence, participants who were assigned male at birth (aOR 1.24 [95% CI 1.20, 1.29]), older (by increasing 5-year increments aOR 1.09 [95% CI 1.09, 1.09]), and/or White (aOR 1.28 [95% CI 1.05, 1.56]) were also more likely to self-report daily PrEP adherence, even when drug concentrations reflected non-daily adherence (Table 5).

**Table 5:** Odds ratio of self-reporting daily adherence among samples with drug concentrations consistent with less-than-daily adherence

	Unadjusted ORs (95% CL)	Adjusted ORs (95% CL)
Sex assigned at birth (vs. female)		
Male	1.26(1.05, 1.51)	1.24(1.20, 1.29)
Age (by increasing 5-year interval)	1.10(1.08, 1.11)	1.09(1.09, 1.09)
Race/ethnicity (vs. white)		
Black	0.83(0.72, 0.95)	0.78(0.64, 0.95)
American Indian or Alaska Native, native Hawaiian, Asian or Other	1.08(0.69, 1.68)	1.09(0.95, 1.26)
Hispanic (vs. non-hispanic)		
Hispanic	0.79(0.34, 1.83)	0.66(0.24, 1.86)
Education level (vs. some high school or less)		
High school graduate or equivalent	0.37(0.12, 1.20)	0.42(0.13, 1.33)
Vocational/trade/technical school	0.16(0.02, 1.29)	0.16(0.02, 1.15)
Come college or 2-year degree	0.42(0.13, 1.34)	0.44(0.16, 1.22)
Finished college	0.48(0.08, 2.69)	0.43(0.10, 1.91)
Master’s or other advanced degree	1.25(0.18, 8.80)	0.83(0.26, 2.58)
Employment status (vs. no employment)		
Part-time employment	0.84(0.51, 1.36)	0.75(0.49, 1.15)
Full-time employment	1.21(0.47, 3.10)	1.23(0.45, 3.32)
Marital Status (vs. married/civil union/legal partnership/primary partner)		
Single/divorced/widowed/Other	1.63(0.92, 2.89)	1.45(0.87, 2.42)
Reports anxiety or depression (vs. denies anxiety and depression)		
Moderately/extremely anxious or depressed	0.35(0.20, 0.60)	0.38(0.23, 0.61)
Illicit drug use (vs. no)		
Yes	0.96(0.77, 1.20)	0.99(0.74, 1.31)

When looking at paired samples that consisted of a self-report of daily adherence, samples from participants who were not fully employed (aOR 2.7 [95% CI 1.31, 5.55]) or who were single/not partnered (aOR 2.33 [95% CI 1.25, 4.34]) were more likely to have drug concentrations consistent with less-than-daily adherence on multivariate analysis. In univariate analysis, participants who were assigned female at birth, identified as Black, or had less than a college education, were also more likely to have drug concentrations consistent with less-than-daily adherence despite self-report of daily adherence, however this finding did not persist after multivariate adjustment. (Table 6).

**Table 6:** Odds ratio of drug concentrations consistent with less-than-daily adherence among samples self-reporting daily adherence

	Unadjusted ORs (95% CL)	Adjusted ORs (95% CL)
Sex assigned at birth (vs. female)		
Male	0.49 (0.28, 0.87)	0.77 (0.38, 1.53)
Age (by increasing 5-year interval)	0.95 (0.84, 1.08)	0.91 (0.79, 1.04)
Race/ethnicity (vs. white)		
Black	3.32 (1.80, 6.13)	2.09 (0.99, 4.38)
American Indian or alaska native, native Hawaiian, Asian or Other	1.71 (0.73, 4.01)	2.01 (0.84, 4.85)
Hispanic (vs. non-hispanic)		
Hispanic	0.57 (0.27, 1.18)	0.57 (0.26, 1.27)
Education level (vs. some high school or less)		
High school graduate or equivalent	0.60 (0.23, 1.53)	0.82 (0.27, 2.44)
Vocational/trade/technical school	0.14 (0.01, 1.34)	0.41 (0.03, 5.36)
Come college or 2-year degree	0.28 (0.11, 0.69)	0.55 (0.18, 1.66)
Finished college	0.25 (0.10, 0.64)	0.76 (0.22, 2.59)
Master’s or other advanced degree	0.11 (0.03, 0.42)	0.31 (0.07, 1.28)
Employment status (vs. no employment)		
Part-time employment	0.51 (0.23, 1.13)	0.49 (0.20, 1.18)
Full-time employment	0.26 (0.14, 0.49)	0.37 (0.18, 0.76)
Marital status (vs. married/civil union/legal partnership/primary partner)		
Single/divorced/widowed/Other	2.08 (1.17, 3.71)	2.33 (1.25, 4.34)
Reports anxiety or depression (vs. denies anxiety and depression)		
Moderately/extremely anxious or depressed	0.97 (0.55, 1.72)	0.85 (0.44, 1.64)
Illicit drug use (vs. no)		
Yes	1.00 (0.57, 1.75)	1.05 (0.55, 2.02)

## Discussion

In HPTN 069/ACTG 5305, 65% of participant samples had plasma drug concentrations consistent with daily medication adherence, with a higher percentage of samples with drug concentrations consistent with daily adherence found among people assigned male at birth (72%) than people assigned female at birth (54%). This is consistent with previous analyses that have found that while PrEP efficacy and adherence has generally been increasing in more recent trials, open-label extensions, and demonstration projects, overall PrEP adherence and challenges differ between the populations studied.

For example, iPrEx, an early randomized controlled trial of daily TDF/ FTC among men who have sex with men (MSM) and transgender women in Latin America, the US, South Africa, and Thailand found adherence was 51% via measurement of detectable TFV in plasma with an overall efficacy rate of 44% [17], in contrast to the PROUD study [18], an open-label randomized wait-listed trial of daily TDF/FTC involving MSM in England which demonstrated improved adherence rates by pill count and an overall efficacy of 86%. Higher PrEP adherence among adults assigned male at birth may be attributable to numerous factors, such as the now established efficacy of oral PrEP, recruitment from populations at elevated risk for HIV with an interest in sexual health services, and the increasing community familiarity with the concept of HIV chemoprophylaxis and its dependence on adherence [2, 8–10]. However, it should be noted that challenges with PrEP adherence and uptake still persist in other populations, such as younger women, as had been described in the VOICE, FEM-PrEP, and HPTN 082 trials [19–21].

Observational and clinical trial data have tended to find higher levels of PrEP adherence among older, educated, and employed US MSM [22–26]. In PrEP demonstration projects [26], the HPTN 067 study [27], and the ATN100 and ATN113 trials [8], older age was associated with better PrEP adherence among MSM. Additionally, a recent analysis of sexual behavior among MSM within our HPTN 069/ACTG 5305 study cohort also found that participants reporting condomless sex had higher rates of plasma drug concentrations classified as adherent [13]. In our analyses, participants who were assigned male at birth, older (compared in groups of increasing 5-year intervals), White, had advanced education, were employed, or partnered/married were more likely to have drug concentrations consistent with daily adherence. The lower study drug concentrations found in participants who identified as female assigned at birth, were younger, Black, or had attained less education may reflect population-level structural and sociodemographic barriers and highlight the role of ongoing adherence monitoring and counseling in clinical trials of new PrEP regimens. Given that rates of new HIV infections are disproportionately occurring in younger persons of color and those who are socially or economically marginalized, PrEP delivery and adherence needs to be tailored to address the unique needs of these populations [28–30]. The findings from this analysis highlight the importance of drug adherence monitoring. Future adherence interventions are needed to address adherence support in culturally responsive ways if all populations are to optimally benefit from PrEP.

An important focus of this analysis evaluated participants who self-reported daily adherence by CASI but had non-daily adherence by drug concentration. Although most samples in this analysis had drug concentrations consistent with daily medication adherence, 11% of participants had paired CASI reports of daily adherence with samples consistent with non-daily drug concentrations. Among samples with self-report of daily adherence, participants who were not fully employed or who were single were found to be more likely to overreport their medication adherence compared to their counterparts, whereas among cases where drug concentrations suggested non-daily adherence, participants who were assigned male at birth, older, or White were found to be more likely to overreport their medication adherence compared to their counterparts. Overestimation of adherence either as a result of social desirability or recall bias is common across medical disciplines and has been observed in many early PrEP efficacy trials and demonstration projects [3, 31, 32]. Despite known discrepancies between drug concentration and self-reported adherence, more recent studies [10, 33–35] have suggested that self-reported PrEP adherence could still be a good proxy for PrEP adherence in the real-world clinical setting.

A limitation of this study that threatens external validity outside of a clinical trial includes the use of blinded study drug regimens that contained three tablets, since currently approved oral PrEP options consist of only a single tablet regimen. To minimize this limitation, all three study drug regimens were formulated to appear identical. Additionally, drug concentrations reflecting adherence from participants in study arms that contained two active study drugs (MVC + TDF; MVC + FTC; TDF + FTC) were generally concordant, suggesting that in this trial the study drug regimen was frequently taken by participants in an all-or-none manner.

Our findings of participants with lower self-reported daily adherence compared to their drug level concentrations may reflect how levels of adherence were measured and classified. This finding contrasts with prior research from PrEP efficacy trials and demonstration projects which had reported that self-report will often overestimate adherence [5–8]. Certain limitations exist in in the currently existing options for measurements of adherence that could account for discrepancies seen between pharmacologic and self-reported indices of adherence, one of which being the timeframes assessed. For instance, although the 3-item self-report questionnaire in this study was framed to assess adherence over the last month, plasma drug concentrations are generally more sensitive to the recency of dosing, reflecting only a day or week of recent dosing. It is known that for pharmacologic measurements of adherence, plasma drug concentrations could be susceptible to intraindividual variability and “white-coat effects”, where participants dose just before their follow-up visit without consistent use [27]. HPTN 069/ACTG 5305 collected plasma drug concentrations and did not collect dried blood spots (DBS) from which intraerythrocytic anabolite concentrations are assessed. Thus, while the presented HPTN 069/ACTG 5305 data reflect short-term objective biomedical measures, it is unclear what longer-term drug dosing patterns were present among study participants. Although it is not clear the degree to which this may have impacted long-term drug concentration results from this trial, the white-coat phenomenon has not been observed to play a large factor when analyzed in other PrEP clinical trials [36–38] that compared plasma drug concentration samples to peripheral blood monocyte concentrations (PBMC), which have a longer half-life and are less susceptible to white-coat effects.

For self-reported measurements, participant responses can be influenced by recall and desirability bias when extended periods of time are required to characterize PrEP adherence. The efficacy of different adherence measures in discriminating between achieving or not achieving daily drug concentrations vary among clinical trials, a finding that may be explained by the differences in the study location and demographic characteristics [39]. The 3-item adherence questionnaire used in this study asked participants to estimate their adherence over the last month, which may be more burdensome than questionnaires asking for recall over a shorter or more recent time period. However, it should be noted that estimation of adherence by 30-day recall has been validated and demonstrated as having lower rates of over-reporting of HIV medication adherence compared to 3-day and 7-day recall [40].

The degree of PrEP adherence that is necessary for adequate protection against HIV is also currently under study. It should also be noted that our analysis focused on measurements of *daily* adherence. However, post-hoc analyses of the iPrEx clinical trial data suggests that drug concentrations consistent with taking four or more doses of TDF/FTC per week is associated with high levels of HIV protection for males assigned at birth and transgender women [41]. This area, as well as questions regarding adherence and efficacy thresholds for on-demand PrEP and drug formulations containing tenofovir alafenamide, requires further study to best minimize limitations in adherence measurements and reporting.

In conclusion, participants in HPTN 069/ACTG 5305 assigned male at birth, who were older, White, college-educated, employed, and/or partnered/married were more likely to have drug concentrations reflecting daily adherence. However, male, older, and White participants were also more likely to overestimate their medication adherence by self-report when drug concentrations suggested less than daily adherence. Additionally, participants who were not fully employed or who were single/not partnered were also more likely to self-report daily adherence despite having drug concentrations consistent with non-daily adherence. Overall, 43% self-reported perfect daily adherence by CASI and 65% of participants had drug concentrations consistent with daily medication adherence. These findings note a discrepancy between self-report of daily adherence and drug concentrations and supports the need for ongoing adherence monitoring and counseling in clinical trials of new PrEP regimens.
